# Structurally novel steroidal spirooxindole by241 potently inhibits tumor growth mainly through ROS-mediated mechanisms

**DOI:** 10.1038/srep31607

**Published:** 2016-08-16

**Authors:** Xiao-Jing Shi, Bin Yu, Jun-Wei Wang, Ping-Ping Qi, Kai Tang, Xin Huang, Hong-Min Liu

**Affiliations:** 1 School of Pharmaceutical Sciences & Collaborative Innovation Center of New Drug Research and Safety Evaluation, Zhengzhou University, Zhengzhou 450001, China

## Abstract

Cancer cells always have increased ROS levels, thus making them more vulnerable to persistent endogenous oxidative stress. The biochemical difference between cancer and normal cells could be exploited to achieve selective cancer cell killing by exogenous ROS-producing agents. Herein we described a structurally novel steroidal spirooxindole by241 and its anticancer efficacy. By241 exhibited potent inhibition against human cancer cells and less toxic to normal cells. By241 concentration-dependently induced apoptosis of MGC-803 and EC9706 cells, accompanied with the mitochondrial dysfunction and increased ROS levels. NAC can completely restore the decreased cell viability of MGC-803 cells caused by by241, suggesting ROS-mediated mechanisms. The expression levels of proteins involved in the mitochondrion-related pathways were detected, showing increased expression of proapoptotic proteins and decreased expression of anti-apoptotic proteins, and activation of caspases-9/-3, but without activating caspase-8 expression. Pretreatment with Z-VAD-FMK partially rescued by241-induced apoptosis of MGC-803 cells. Additionally, by241 inhibited mTOR, activated p53 and its downstream proteins, cleaved MDM2 and PI3K/AKT as well as NF-κB signaling pathway. *In vivo* experiments showed that by241 did not have significant acute oral toxicity and exerted good anticancer efficacy against MGC-803 bearing mice models. Therefore, by241 may serve as a lead for further development for cancer therapy.

Reactive oxygen species (ROS) including hydrogen peroxide (H_2_O_2_), superoxide anion (O_2_^−^), and hydroxyl radical (HO∙) are formed through the incomplete reduction of oxygen in normal physiological processes (e.g. the oxidative metabolism). Cellular ROS can be generated through multiple mechanisms, mainly from the mitochondrial respiratory chain and partly from potential interactions with exogenous ROS sources such as UV light, ionizing radiation, inflammatory cytokines, carcinogens, etc^1^. ROS play essential roles in maintaining vital biological functions through regulating many signaling pathways (e.g. MAPK, PI3K, Nrf2 and Ref1-mediated signaling pathways)^2^ and have also proven to be able to promote cell proliferation and differentiation under threshold levels^3^. ROS, however, act as a double-edged sword in living cells^4^. The accumulation of ROS to excessive levels can result in irreversible oxidative damage to lipids, proteins and DNA. Therefore, controlling ROS under critical threshold levels by cellular redox homeostasis is crucial for normal cells to maintain their growth and survival. Compared to normal cells, cancer cells have higher demand on the mitochondrial respiratory chain to generate more ATP for their rapid growth and differentiation, thus inevitably making cancer cells have high levels of endogenous oxidative stress. Increasing evidence has shown that the aggressiveness of tumors and poor prognosis always correlate with increased ROS levels in cancer cells^5^. Increased ROS levels, on the other hand, make cancer cells more vulnerable to persistent oxidative stress caused by ROS-generating agents^6^. The different redox states between normal and cancer cells would provide an opportunity to selectively induce cancer cell death^7^. To date, a large number of ROS-generating agents such as procarbazine have been identified, relying on ROS production for their anticancer efficacy^8^.

Steroids, an important class of polycyclic compounds, are prevalent in nature and well known for their diverse and profound biological activities, as well as the abilities of maintaining normal biological functions in living organisms^9^,^10^,^11^. Chemical modifications on steroids have long been pursued to generate structurally novel and/or biologically important molecules, especially the incorporation of heterocycles into the steroid core. To date, a large number of biologically interesting steroids have been identified and some of them are being used in clinic for the treatment of diseases^12^. Two representative examples are abiraterone^13^ and galeterone^14^ bearing the pyridine and benzimidazole heterocycles at the C-17 position, respectively (Fig. 1), which are currently used in clinic for the treatment of advanced prostate cancers as androgen synthesis inhibitors. Dehydroepiandrosterone (DHEA), an endogenous steroid secreted by the adrenal cortex, is able to inhibit proliferation of human cancer cells both *in vitro* and *in vivo* through multiple mechanisms^15^,^16^. Besides, DHEA, as the dietary supplement, has been used as the anti-aging hormone since 1980s. All these studies may suggest that DHEA has anticancer potential and is less toxic to normal cells and therefore could be used as a starting point for developing potent steroid-based anticancer agents. Based on these considerations, we previously designed and synthesized a large number of DHEA-based steroidal derivatives and tested their anticancer properties against human cancer cells of different origins^17^,^18^,^19^,^20^,^21^,^22^,^23^. Some of them exhibited potent anticancer activity. From our in-house steroid library, one structurally novel steroidal derivative (named **by241**, Fig. 1) stood out with favorable anticancer efficacy, featuring a spiro-cyclic oxindole scaffold attached to the steroid nucleus^24^,^25^,^26^. In the present study, we would like to report the anticancer properties of this shortlisted compound and its possible mechanisms of action. By241 may serve as a template for developing more potent anticancer agents for cancer therapy.

## Results and Discussion

### Synthesis of by241

**Figure 1:**
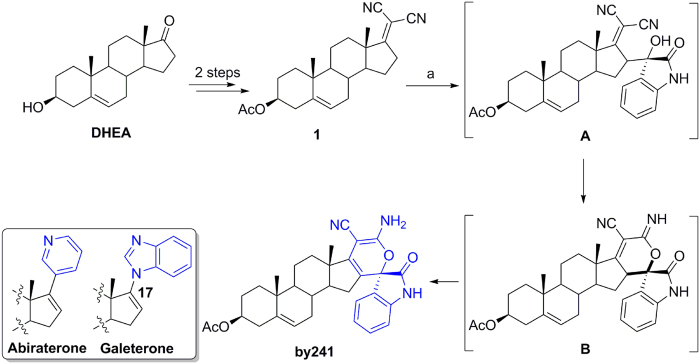
Chemical structures of abiraterone and galeterone and synthesis of by241. Reagents and conditions: (**a**) Isatin, Et_3_N, EtOH/H_2_O (1/1), rt.

As shown in Fig. 1, the synthesis of by241 involved the vinylogous aldol reaction of the steroidal dicyanoalkyene **1** with isatin, followed by intramolecular cyclization and isomerization sequence in the presence of Et_3_N, affording by241 in 88% yield under mild conditions. One C-C single bond, one C-O bond, as well as one quaternary carbon center were formed in this one-pot reaction. It should be noted that we obtained intermediate **B** from compound **1** when this kind of reaction was performed in EtOH, not the EtOH/H_2_O mixture using the sterically hindered DBU as the base. The structural characteristics of by241 lie in the spiro-fused oxindole and 2*H*-pyran scaffolds connected through a quaternary carbon center. Compound **1** was efficiently prepared from DHEA within two steps following our previously reported methods^18^.

### Cytotoxicity of by241 against human cancer and normal cell lines

With by241 in hand, we next tested its cytotoxicity against several human cancer cell lines of different origins using the MTT assay. The well-known anticancer drug 5-fluorouracil (5-FU) was selected as the reference drug to compare the *in vitro* and *in vivo* anticancer potency. Human normal liver cell line (L-02) and human normal esophageal cell line (Het-1A) were chose to investigate the toxicity and selectivity of by241. The IC_50_ values of by241 and 5-FU against tested cancer cell lines and human normal cell lines are summarized in Table 1. Generally, by241 had broad-spectrum anticancer activity, showing favorable inhibition against the tested cancer cell lines (IC_50_ < 6.5 μM). Also, by241 was more potent than 5-FU and less toxic to human normal cells (IC_50_ > 20 μM), indicating good selectivity. Specifically, by241 exhibited excellent inhibition against human gastric cancer cells (MGC-803 and BGC-803) with the IC_50_ values of 2.77, 1.18 μM respectively. By241 inhibited growth of L-02 with an IC_50_ value of 21.80 μM, showing around 5- and 8-fold selectivity toward SMMC-7721 (IC_50_ = 4.83 μM) and ZIP77 (IC_50_ = 2.71 μM), respectively. By241 also displayed similar selectivity to Het-1A (IC_50_ = 20.15 μM) over other human esophageal cancer cells (EC109, EC9706, and KYSE450). For normal liver L-02 cells, by241 was about 5-fold less toxic than 5-FU, suggesting a relatively low toxicity of by241 (IC_50_ = 21.80 μM *vs*. 4.37 μM). Additionally, a slight difference in inhibiting growth of sub-types of gastric cancer cells has also been observed (IC_50_ = 2.77 and 1.18 μM, respectively against MGC-803 and BGC-803).

**Figure 2:**
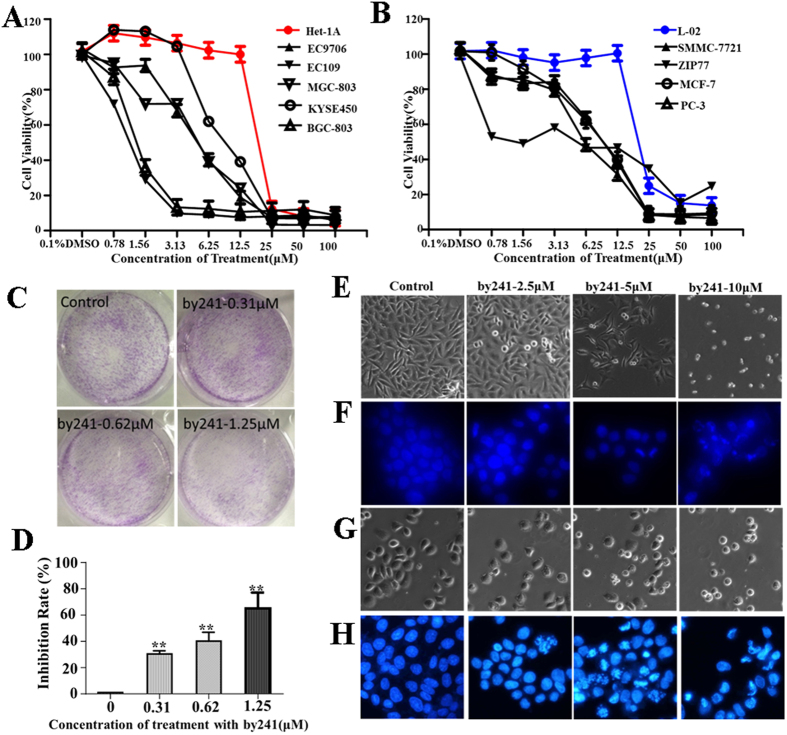
Antiproliferative effects of by241 on selected human cell lines. (**A**) and (**B**) Human cells were treated with different concentrations of by241 for 72 h. Cell viability determined by MTT assay; (**C**) Representative images of MGC-803 cells colonies after treatment with various concentrations (0.31, 0.62 or 1.25 μM) of by241 for 12 days; (**D**) Quantitative analysis of the colony formation inhibition rate in Fig. 1C. Apoptosis related morphology and nuclear condensation of MGC-803 (**E**,**F**) induced by by241. Apoptosis related morphology and nuclear condensation of EC9706 (**G**,**H**) induced by by241. Cells were cultured with different concentrations of by241 (2.5, 5 and 10 μM) for 24 h and detected using the Hoechst 33258 assay. Data are presented as the mean  ±  SD of three independent experiments. **P < 0.01 was considered statistically compared to control.

After exposure to by241 at different concentrations for 72 h, the cell viability of cancerous cells and normal cells was decreased gradually with the concentration increase of by241, as demonstrated in Fig. 2A,B. For normal cells L-02 and Het-1A, the cell viability was almost unchanged even at high concentration (12.5 μM). In contrast, the viability of cancer cells dropped significantly, especially the BGC-803 and MGC-803 cells. The difference between normal cells and cancerous cells in cell viability also showed low toxicity and good selectivity of by241. At higher concentrations (>12.5 μM), the cell viability of normal cells declined substantially, suggesting the toxicity of by241 to normal cells at high concentrations.

The clonogenic assay represents an indirect estimation of neoplastic transformation. As shown in Fig. 2C, smaller and fewer colonies were formed when MGC-803 cells were treated with increasing concentration of by241, the inhibition rate of colony formation was about 60% when treated with by241 at 1.25 μM for 12 days (Fig. 2D). Besides, morphological changes of MGC-803 and EC9706 cells such as rounding up and cell debris were observed, especially at high concentrations, after being incubated with by241 for 24 h at different concentrations (0, 2.5, 5, 10 μM) (Fig. 2E,G). After staining with Hoechst 33258, remarkable nuclear changes of MGC-803 and EC9706 cells including the chromatin condensation, nuclear fragmentation and condensation were also observed (Fig. 2F,H).

### Cell apoptosis induced by by241

**Figure 3:**
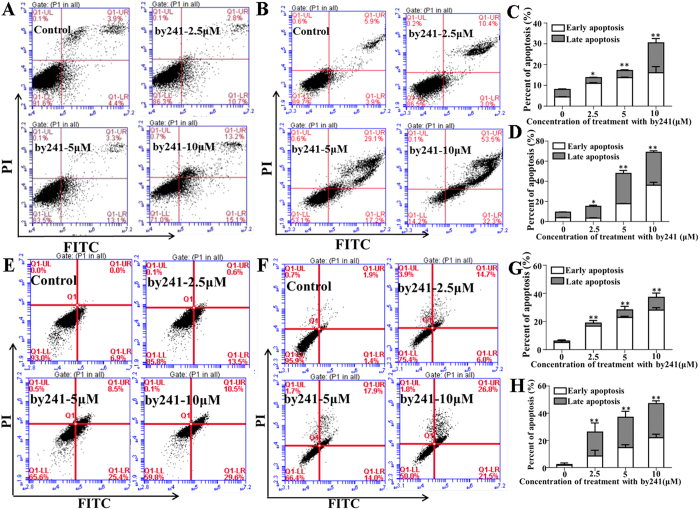
By241 induced cell apoptosis. Apoptosis cells were detected using the Annexin V-FITC/PI double staining after exposure to by241 at different concentrations (0, 2.5, 5, 10 μM) and analyzed by flow cytometry and the apoptotic cell rate was analyzed after Annexin V-FITC/PI staining. (**A**,**B**) MGC-803 were treated for 12 h or 24 h; (**E**,**F**) EC9706 were treated for 12 h or 24 h; (**C**,**D**) Statistical analysis of apoptotic cells of (**A**,**B**); (**G**,**H**) Statistical analysis of apoptotic cells of (**E**,**F**); data are presented as the mean ± SD of three independent experiments. *P < 0.05 and **P < 0.01 were considered statistically compared to control.

Above studies showed that by241 potently inhibited growth of cancer cells and induced morphological changes of MGC-803 and EC9706 cells in a concentration-dependent manner. These studies suggest that by241 should be capable of inducing apoptosis of cancer cells. Therefore, we performed the flow cytometric analysis of MGC-803 and EC9706 cells using the Annexin V-FITC and propidium iodide (PI) double staining after being incubated with by241 at different concentrations (0, 2.5, 5, 10 μM) for 12 or 24 h. As shown in Fig. 3A–D, by241 markedly induced apoptosis of MGC-803 cells in a concentration-/time-dependent manner, the late apoptosis in particular. Specifically, after treatment with by241 for 12 h, the apoptotic cells accounted for 28.3% at 10 μM, higher than that of the control group (Fig. 3A,C). More evidently, for the 24 h group, the apoptotic cells amounted to 85.8%, significantly higher than that of the control group (Fig. 3B,D). Besides, by241 also induced apoptosis of EC9706 cells in a concentration-/time-dependent manner (Fig. 3E–H). After treatment of EC9706 cells for 12 h at 10 μM, the percentage of apoptotic cells was 40.1%, significantly higher than the control group, but slightly lower than that of the group treated for 24 h at the same concentration (Fig. 3F,H).

### By241 induced cell death through ROS-mediated mechanisms

The favorable potency of by241 toward cancer cells promoted us to investigate the potential mechanisms of action. More recently, Banerjee *et al*. designed a novel fluorescent cancer cell detector (named Is-Bet A) by combining the bis-arylidene oxindole and natural betulinic acid through an amino propyl-linker, in which betulinic acid, structurally belonging to steroids, acted as the ROS-generator, while the bis-arylidene oxindole served as a fluorophore for detection, thus achieving simultaneous detection and killing of cancer cells^27^. Mounting evidence has showed that steroid hormones can increase the ROS production in mitochondria^28^,^29^,^30^,^31^,^32^. Among them, DHEA has been proved to be able to increase ROS formation by inhibiting segment I of the respiratory chain^33^. Oliveira and co-workers reported that *Uncaria tomentosa* extract containing oxindole alkaloids triggered apoptosis of HT29 cells through ROS-mediated caspase activation and DNA repair^34^. Above studies suggest that both steroids and oxindole containing compounds could serve as ROS-generating agents for cancer therapy. From the structural point of view, we speculate that our target molecule by241 incorporating oxindole and steroid nucleus potentially induced cell death through ROS-mediated mechanisms.

**Figure 4:**
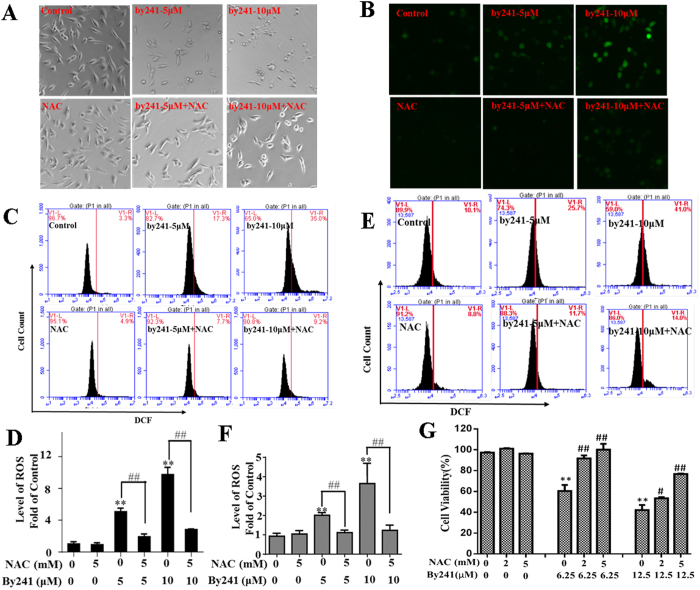
The role of ROS in by241-induced cancer cells death. After pretreatment with or without 5 mM NAC for 2 hours, MGC-803 cells were treated with or without by241 for 24 hours, then the apoptosis related morphology (**A**) and the intracellular ROS was detected by fluorescence microscope (**B**); After pretreatment with or without 5 mM NAC for 2 hours, MGC-803 cells (**C**) or EC9706 cells (**E**) were treated with or without by241 for 24 hours, then the intracellular ROS was detected by flow cytometry; (**D**,**F**) Statistical analysis of ROS levels of (**C**,**E**); (**G**) After pretreatment with or without 2 mM or 5 mM NAC for 2 hours, MGC-803 cells were treated with or without by241(6.25, 12.5 μM) for 24 hours, cell viability were detected by the MTT assay. Data are presented as the mean ± SD of three independent experiments. **P < 0.01 and ^##^*P* < 0.01 were considered statistically compared to corresponding control. *P < 0.05, **P < 0.01 compared to control; ^##^*P* < 0.01 NAC+ by241-5 μM compared to by241–5 μM or NAC+ by241–10 μM compared to by241–10 μM.

As shown in Fig. 2E–H, by241 induced remarkable morphological changes of MGC-803 and EC9706 cells, especially at high concentrations. Interestingly, treatment of MGC-803 cells with *N*-acetyl-*L*-cysteine (NAC, 5 mM) rescued the by241-induced morphological changes of MGC-803 cells (Fig. 4A), suggesting that by241 probably induced cell death through elevating cellular ROS levels. We then used the DCFH-DA assay to determine the ROS levels in MGC-803 cells, after treatment with by241 at different concentrations (0, 5, 10 μM), following DCFH-DA treatment for 30 min, the green fluorescence was analyzed using an inverted fluorescence microscope. As shown in Fig. 4B, By241 concentration-dependently enhanced the green fluorescence intensity, the percentage of cells with green fluorescence amounted to 41.0% when treated at 10 μM, significantly higher than that of the control group (Fig. 4C), and NAC can decrease the green fluorescence intensity in MGC-803 cells induced by by241, the percentage of cells with green fluorescence was only 14.0% when treated with by241 (10 μM) and NAC (5 mM), slightly higher than that of the control group (8.8%). For EC9706 cells, the percentage of cells with green fluorescence intensity amounted to 35% (around 9-fold increase relative to the control) when treated with by241 at 10 μM, which then decreased to 9.2% when treated with by241 (10 μM) and NAC (5 mM) (Fig. 4E,F).

NAC has been proved to be able to attenuate ROS generator-induced cancer cell death^35^,^36^. To investigate whether NAC can attenuate by241-induced MGC-803 cell death, NAC alone and in combination with by241 were used to explore the cytoprotective effect of NAC against the by241-induced MGC-803 cell death. As shown in Fig. 4G, NAC alone had no effect on the cell viability of MGC-803 cells even at the concentration of 5 mM, while NAC was found to be able to completely restore the decreased cell viability of MGC-803 cells caused by by241 at 6.25 μM. Around 80% of cell viability was observed when MGC-803 cells were treated with 12.5 μM and 5 mM of NAC (Fig. 4G). This result supported the hypothesis that by241 induced cell death mainly through ROS-mediated mechanisms.

**Figure 5:**
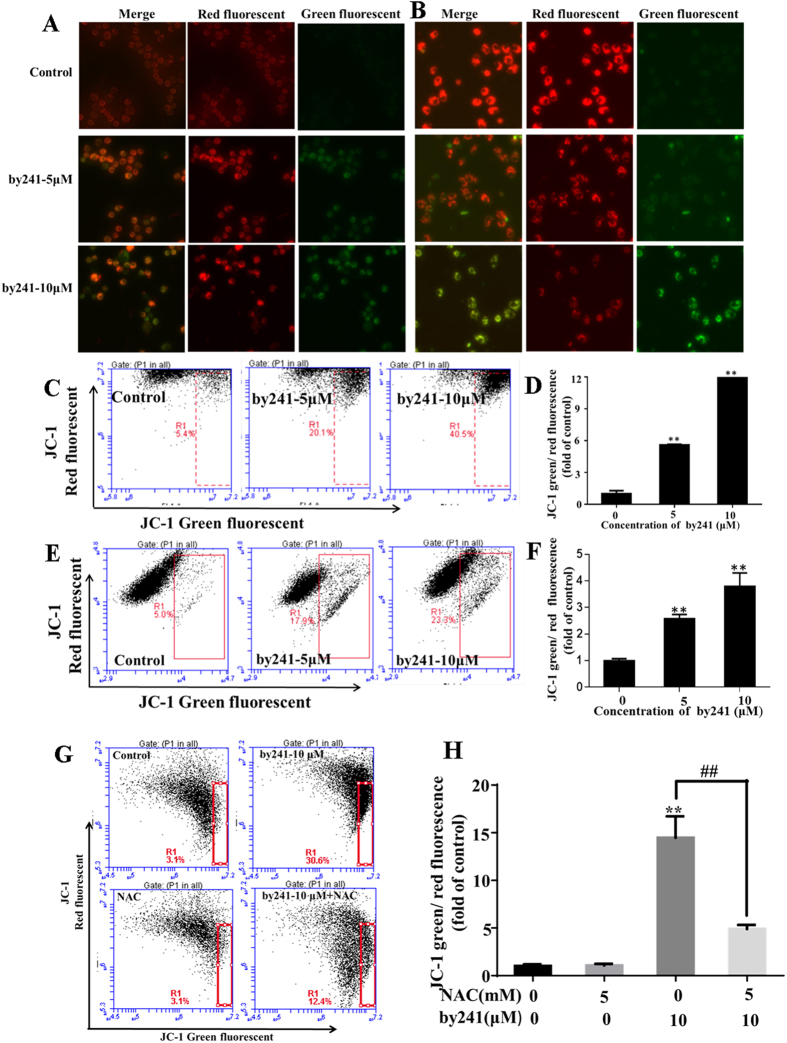
By241 induced mitochondrial dysfunction. Cells were treated with 5, 10 μM of by241 for 24 hours, JC-1 staining image of (**A**) MGC-803 and (**B**) EC9706 cells were detected by fluorescence microscope, JC-1 staining red and green fluorescence intensity of (**C**) MGC-803 and (**E**) EC9706 cells analyzed by flow cytometry. (**D**,**F**) Quantitative analysis of the ratio of green/red fluorescence in Fig. 5C,E. (**G**) MGC-803 Cells were pretreated with or without 5 mM NAC for 2 hours, and then incubated with the by241 for another 24 hours, JC-1 staining red and green fluorescence intensity was analyzed by flow cytometry. (**H**) Quantitative analysis of the ratio of green to red fluorescence in Fig. 5G. Data are presented as the mean ± SD of three independent experiments. **P < 0.01 were considered statistically significant compared with the controls, ^##^*P* < 0.01 NAC+by241-10μM compared to by241–10 μM.

To date, the relationship between mitochondrial damage and ROS changes is not clearly understood. It has been widely accepted that mitochondrial dysfunction is always associated with increased ROS production, accompanied with changes of mitochondrial membrane permeability, resulting in the loss of mitochondrial membrane potential (MMP, *ΔΨm*) and activation of downstream caspases^35^. Hence, in this study, the *ΔΨm* was measured using the JC-1 dye to evaluate the by241-induced mitochondrial dysfunction (Fig. 5). As shown in Fig. 5A,B, by241 concentration-dependently increased the green fluorescence intensity in MGC-803 cells and EC9706 cells, indicating the by241-induced translocation of JC-1 dye from the mitochondria to the cytoplasm. The changes of the green/red fluorescence intensity in MGC-803 and EC9706 cells were then quantitatively analyzed using the flow cytometry analysis. As shown in Fig. 5C,D, a concentration-dependent decrease of MMP represented by the green/red fluorescence intensity was observed. At 10 μM, the fold of green/red fluorescence intensity of MGC-803 cells with the green fluorescence increased 12.0 fold. Similarly, the fold of green/red fluorescence intensity labeled EC9706 cells amounted increased 3.9 fold when treated with by241 at 10 μM, showing a concentration-dependent increase (Fig. 5E,F). Furthermore, the decreased MMP caused by by241 in MGC-803 could mostly reversed by NAC (Fig. 5G,H).

**Figure 6:**
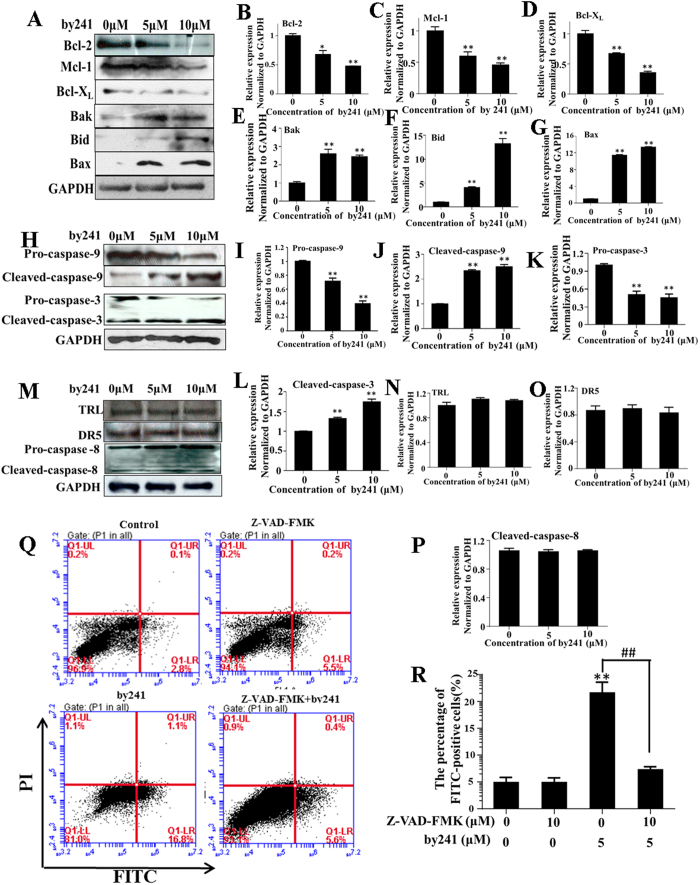
Expression changes of apoptosis related proteins induced by by241. (**A**) By241 induced expression changes of Bcl-2 family (Bcl-2, Mcl-1, Bcl-XL, Bak, Bid, Bax) proteins; (**B**–**G**) Statistical analysis of Bcl-2 family (Bcl-2, Mcl-1, Bcl-XL, Bak, Bid, Bax) proteins expression change induced by by241; (**H**) By241 induced caspase-3 and caspase-9 activation; (**I**–**L**) Statistical analysis of pro-caspase-3, cleaved caspase-3, pro-caspase-9 and cleaved caspase-9 expression levels; (**M**–**P**) By241 did not change the expression of TRL, DR5, and cleaved caspase-8. (**Q**) Effect of pan caspase inhibitor Z-VAD-FMK significantly attenuated by241-induced apoptosis MGC-803 cells; (**R**) Statistically analysis of the FITC-positive cells. Data are presented as the mean ± SD of three independent experiments. *P < 0.05, **P < 0.01 compared to control; ^##^*P* < 0.01 Z-VAD-FMK+by241-5 μM compared to by241-5 μM.

Next, we examined the expression levels of mitochondria related Bcl-2 family proteins including pro-apoptotic proteins (Bax, Bid and Bak) and anti-apoptotic proteins (Bcl-2, Mcl-1 and Bcl-X_L_). As shown in Fig. 6, after treatment with by241, expression levels of pro-apoptotic proteins increased concentration-dependently (Fig. 6E–G), particularly the Bid and Bax. Activated Bid is believed to be able to interact with Bax and then promotes the insertion of Bax into the mitochondrial outer membrane^37^. Besides, Bax and Bak are also able to promote the release of cytochrome c and other pro-apoptotic factors from the mitochondria, ultimately leading to activation of caspases by inducing the opening of mitochondrial voltage-dependent anion channel (VDAC) and/or forming the oligomeric pore MAC. Expressions of the anti-apoptotic proteins Bcl-2, Mcl-1 and Bcl-X_L_ decreased correspondingly (Fig. 6B–D).

Three major apoptosis-associated pathways to caspase activation have been identified and ordering of caspases involved is currently relatively well understood^38^,^39^. Above studies have shown that by241 markedly increased expression of pro-apoptotic proteins Bid, Bax, and Bak, which have been proved to be able to activate expressions of downstream caspases. Herein, expression levels of three main caspases, namely the caspase-8, caspase-9, and caspase-3, were measured using the Western blot analysis. As shown in Fig. 6H, by241 concentration-dependently resulted in activation of pro-caspases-9 and -3, leading to increased expression of cleaved caspase-9 and caspase-3. Several reports described that ROS was capable of inducing cell death through activating caspase-8^40^,^41^,^42^. However, in this study, the expression of cleaved caspase-8, DR5 and TLR was almost unchanged. Associated with overexpressed Bid (as shown in Fig. 6M), we reason that by241 induced apoptosis of MGC-803 cells in part through the mitochondria-related caspase-9/caspase-3 intrinsic pathways, not the membrane death receptor-mediated extrinsic pathways or the caspase-8/Bid/Bax pathway.

**Figure 7:**
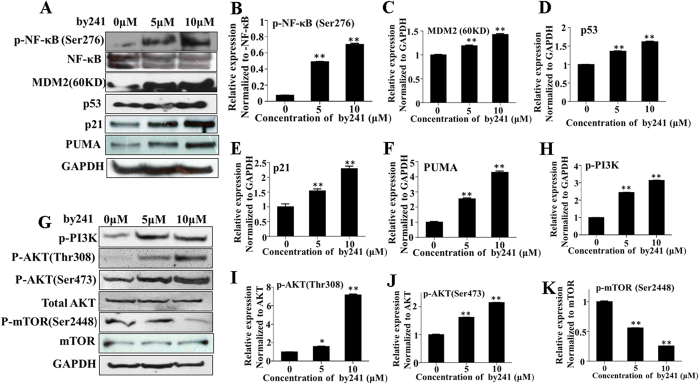
Expression changes of key proteins involved in ROS mediated pathways. (**A**) Expression analysis of p-NF-κB, MDM2, p53, p21 and PUMA in by241-treated MGC-803 cells. Western blot of protein extracted from MGC-803 cells following 24 h treatment with by241 (5 μM and 10 μM), a representative result of 3 independent experiments is shown. (**B**–**F**) Statistical analysis of p-NF-κB, MDM2, p53, p21 and PUMA proteins expression change induced by by241; (**G**) Expression analysis of p-PI3K, p-AKT (Thr308), p-AKT (Ser473), AKT, p-mTOR (Ser2448) and mTOR in by241-treated EC109 cells. (**H**–**K**) Statistical analysis of p-PI3K, p-AKT (Thr308), p-AKT (Ser473) and p-mTOR (Ser2448) proteins expression changes induced by by241; Data are presented as the mean ± SD of three independent experiments. *P < 0.05, **P < 0.01 were considered statistically significant compared with the controls.

Furthermore, as shown in Fig. 6Q,R, pretreatment of MGC-803 cells with pan caspase inhibitor Z-VAD-FMK led to a decrease of FITC-positive cells (Apoptotic cells induced by by241). Specifically, the apoptotic cells for the by241-treated group accounted for about 22%, while the percentage of apoptotic cells after treatment with by241 and Z-VAD-FMK decreased to around 7%, but still slightly higher than that of cells treated with Z-VAD-FMK alone (about 5%, Fig. 7R). These results revealed that pan caspase inhibitor Z-VAD-FMK cannot prevent by241-induced apoptosis completely, suggesting that apart from the mitochondria-mediated apoptotic pathways, simulation of other signaling pathways and/or key proteins involved in the ROS-mediated pathways by by241 also contributed to MGC-803 cell death.

Apart from the mitochondria-mediated apoptotic pathways, mounting evidence has shown that ROS can regulate many other signaling pathways such as p53^43^,^44^,^45^,^46^,^47^. Activated p53 then induces expression of downstream transcriptional proapoptotic proteins such as Bax and PUMA^48^, ultimately leading to apoptosis of cancer cells. We next examined expression changes of key proteins related to ROS-mediated apoptosis induced by by241. As shown in Fig. 7, treatment with by241 increased expression of p53, which then transcriptionally activated its downstream proapoptotic proteins such as Bax (Fig. 6A) and PUMA (Fig. 7A). p53 also induced expression of p21 (Fig. 7A), which, as the cyclin-dependent kinase (CDK) inhibitor, can arrest cell cycle at G1 phase^49^,^50^ and is also able to inhibit cancer cell growth^51^,^52^. Also, by241 concentration-dependently cleaved MDM2, generating a 60KD MDM2 fragment (Fig. 7A), which at least in part could be explained by the caspase-3 activation. It has been reported that caspase-3 can cleave the MDM2 oncoprotein during p53-mediated apoptosis^53^,^54^.

The transcription factor NF-κB plays vital roles in regulating cell differentiation, responses to oxidative stress and apoptosis through controlling expression of related genes^55^. The crosstalk between NF-κB and p53 is regulated by the relative levels of each transcriptional factor. ROS-induced p53 activation therefore could down-regulate NF-κB, accompanied with increased expression of phosphorylated NF-ĸB (*p*-NF-ĸB) (Fig. 7A). To date, a large number of natural and synthetic small molecules, as well as peptides have been identified as NF-κB inhibitors^56^. However, the therapeutical effect of NF-κB inhibitors as anticancer agents is controversial. Recently, Ryan *et al*. described that inhibition of NF-κB in wild-type p53 retaining tumors may cause a diminished therapeutical response^57^.

Besides, ROS also activated the PI3K/AKT pathways^58^, leading to increased expression of key proteins such as PI3K and AKT (Fig. 7G). mTOR, as a conserved serine/threonine kinase, is crucial in regulating cell survival and proliferation. As shown in Fig. 7G, the expression of *p*-m-TOR was down-regulated, which could be achieved through the ROS-JNK-p53 pathway^47^ or by activating AMPKα^59^.

### *In vivo* acute oral toxicity and anti-tumor activity of by241

**Figure 8:**
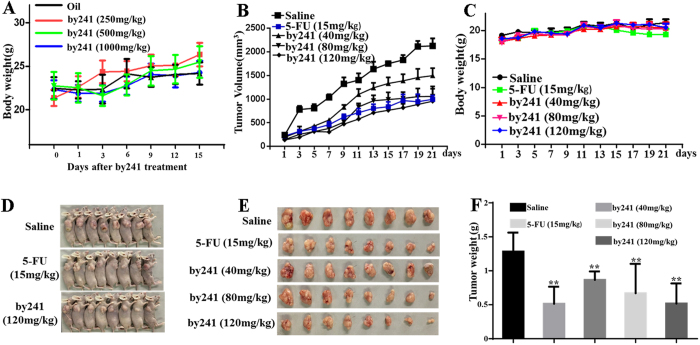
The acute oral toxicity of by241 on mice and antitumor efficacy in MGC-803 bearing nude model. (**A**) Body weight of mice in 15 days after oral treatment with by241 (250, 500 or 1000 mg/kg). MGC-803 cells were transplanted subcutaneously to the BALB-C nude mice and subjected to by241 (40, 80 and 120 mg/kg), 5-FU (15 mg/kg) and saline were used as the negative control for 21 days. (**B**) Tumor size and (**C**) body weight measurements every 2 days from MGC-803 mice after by241 administration. (**D**) Photographs of mice in saline group, 5-FU group and by241 (120 mg/kg) group and (**E**) Photographs of tumors in each group. (**F**) Comparison of the final tumor weight in each group after 21-day treatment. Data are presented as means ± SD. **P < 0.01 was considered statistically significant compared with the negative control.

Due to the favorable potency in inhibiting growth of cancer cells *in vitro* and low toxicity to normal cells, we then evaluated the *in vivo* acute oral toxicity of by241 on mice, which may provide a guideline for selecting doses for further *in vivo* experiments. As shown in Table 2, no severe side effects or mortality in test groups were observed even at the dose of 1000 mg/Kg. Animals did not show significant abnormal signs, behavioral changes, and water or food consumption during observation. As shown in Fig. 8A, body weight changes of animals treated with by241 relative to the control was not remarkable. Furthermore, no significant changes or lesion in the viscera of test animals were observed in autopsy experiments.

The favorable *in vitro* potency and low toxicity of by241 observed promoted us to investigate the *in vivo* anti-tumor potency. MGC-803 cells were injected in the paw subcutaneous of the mice. Three days later, by241 were intragastrically administered to the mice daily at different doses (40, 80 and 120 mg/Kg) using 5-FU (15 mg/Kg), saline as the positive and negative controls, respectively. During the 21-day treatment, the mouse body weight was monitored every 2 days. As shown in Fig. 8C, compared to the controls, the mice administered daily with by241 even at 120 mg/Kg did not show any body weight loss, while the body weight of mice treated with 15 mg/Kg 5-FU daily declined gradually during the last 4-day treatment, showing less toxicity of by241 than 5-FU after repeated 21-day treatment at high doses. Besides, as shown in Fig. 8B, by241 exhibited favorable anticancer efficacy at 40 mg, 80 and 120 mg/kg, the corresponding tumor volume for the by241 (120 mg/kg) treatment group was significantly smaller than that of the saline group. At the end of the experiment, tumor volume was 2126 mm^3^ in the control group, while tumor volume in the treatment groups was 1247 mm3, 1060 mm^3^ and 957 mm^3^ at by241 doses of 40 mg/kg, 80 mg/kg and 120 mg/kg, respectively. The tumor volume of the 5-FU group was 822 mm^3^. Compared to the control group treated with saline (1.3 g), the average tumor weight of mice treated with by241 at 40, 80 and 120 mg/kg decreased to around 0.87 g, 0.67 g and 0.52 g, respectively (Fig. 8E,F), accounting for a 32.3%, 47.7% and 59.6% reduction in tumor weight. And the average tumor weight of the 5-FU group was 0.51 g, showing 59.8% decrease in tumor weight. The favorable *in vitro* and *in vivo* anticancer potency and the absence of acute oral toxicity even at high doses (Table 2 and Fig. 8A) warrant its further development for cancer therapy. As shown in Table 1, by241 was more potent than 5-FU against tested cancer cells, but the inferior *in vivo* anticancer activity of by241 could be attributed to the relatively poor bioavailability of by241.

## Conclusions

In summary, we, for the first time, synthesized the structurally novel steroidal spirooxindole derivative by241 from the corresponding dicyanoalkyene **1** and isatin through the base-promoted cascade reactions, in which one C-C single bond, one C-O bond, as well as one quaternary carbon center were formed. Structurally, by241 shared the same steroid nucleus with anticancer drugs abiraterone and galeterone and also featured a biologically important spirooxindole scaffold.

Compared to 5-FU, by241 exhibited more potent inhibition against the tested cancer cells (EC109, EC9706, KYSE450, MGC-803, BGC-803, SMMC-7721, ZIP77, MCF-7 and PC-3) and was less toxic to human normal cells (Het-1A and L-02), indicating a good selectivity toward cancer cells and normal cells. By241 concentration-dependently inhibited the colony formation, induced typical morphological changes and remarkable apoptosis of MGC-803 cells. Moreover, treatment of MGC-803 and EC9706 cells with by241 resulted in the mitochondrial dysfunction, accompanied with increased ROS levels and decrease of *MMP*. Interestingly, ROS scavenger NAC was found to be able to completely restore the decreased cell viability, the increased ROS and the decrease of *MMP* of MGC-803 cells caused by by241, suggesting the ROS-mediated mechanisms for observed cell death. The expression levels of key proteins involved in the mitochondria-mediated pathways such as Bcl-2 family members and downstream proteins were detected using the Western blot analysis, showing increased expression of proapoptotic proteins such as Bid, Bax and Bak, decreased expression of anti-apoptotic proteins (Bcl-2, Mcl-1 and Bcl-X_L_), and activation of caspase-9/-3, finally leading to apoptosis. However, by241 did not affect expression of caspase-8, DR5 and TLR. All these data suggest that by241 induced apoptosis of MGC-803 cells through the mitochondria-mediated caspase-9/caspase-3 intrinsic pathways, not the caspase-8 involved pathways such as the membrane death receptor-mediated extrinsic pathway and the caspase-8/Bid/Bax pathway. More interestingly, pretreatment of MGC-803 cells with pan caspase inhibitor Z-VAD-FMK decreased the percentage of apoptotic cells induced by by241, which implies that by241 induced apoptosis of MGC-803 cells through multiple mechanisms, not limited to the intrinsic apoptotic pathway. Further mechanistic studies were carried out focusing on other ROS-related pathways, showing that by241 also activated p53 and its downstream proteins such as PUMA and p21, inhibited NF-κB and mTOR, cleaved MDM2, and activated PI3K/AKT signaling pathway. These ROS-mediated pathways also contributed to the cell death of MGC-803. Taken together, by241 induced cell death through ROS-mediated multiple mechanisms. Further *in vivo* experiments showed that by241 did not have significant acute oral toxicity on mice even at high dose and exerted good *in vivo* anticancer efficacy. Therefore, by241 may serve as a lead for developing potent steroid-based anticancer drugs. Further work will be focused on developing more potent by241 derivatives with improved bioavailability through further SARs studies. Also, in combination with other drugs targeting the redox adaptation of cancer cells to the increased oxidative stress will be carried out to overcome the potential drug resistance.

### Experimental section

#### Synthesis of by241

Compound **1** was efficiently prepared from DHEA in two steps following our previously reported protocols^17^,^18^,^19^. Therefore, the synthetic details of compound **1** and associated NMR data were not given here. Procedure for the synthesis of by241 from compound **1** is described herein: To a solution of compound **1** (1.0 mmol) and isatin (1.0 mmol) in a mixture of EtOH (5 mL) and H_2_O (5 mL) was added triethylamine (TEA, 2.0 mmol) dropwise at room temperature. Upon completion of this reaction (monitored by TLC), the solvent was removed under vacuum, the resulting residue was then subjected to recrystallization in EtOAc, affording the by241 as a brownish red solid. HPLC purity: >95% (AGILENT-C18 5 μm 4.6 × 250 mm, MeOH/H_2_O 80%/20%, 1 mL/min, UV: 210 nM, 30 °C); Yield: 88%, m. p. 222.5-223.8 °C; ^1^H NMR (400 MHz, CDCl_3_) δ 9.28 (s, 1H), 7.32 (d, *J* = 7.2 Hz, 1H), 7.07 (brs, 1H), 6.95 – 6.82 (m, 2H), 6.27 (brs, 1H), 6.20 (d, *J* = 7.2 Hz, 1H), 5.43 (d, *J* = 4.4 Hz, 1H), 4.73 – 4.50 (m, 1H), 2.05 (s, 3H), 1.06 (s, 3H), 1.02 (s, 3H); ^13^C NMR (100 MHz, CDCl_3_) δ 170.54, 170.45, 167.42, 147.78, 141.17, 140.19, 130.35, 130.22, 124.45, 123.54, 121.42, 116.50, 109.58, 105.91, 73.67, 50.64, 49.53, 47.83, 38.04, 36.74, 36.62, 35.15, 32.98, 31.94, 30.92, 27.63, 21.42, 19.33, 16.25; HRMS (ESI): *m/z* calcd for C_32_H_36_N_3_O_4_ (M+H)^+^, 526.2706; found, 526.2703.

#### Biological evaluation

##### Reagents, antibodies and animals

By241 was dissolved in DMSO to make a 100 mM stock solution. Working concentrations were created by diluting the stock solution in RPMI-1640 media containing 10% Fetal Bovine Serum. RPMI-1640 and Fetal Bovine Serum were obtained from Hyclone Laboratories(Utah, USA). 5-Fluorouracil (5-FU) was purchased from the Shanghai Xudong Haipu Pharmaceutical Co.Ltd.(Shanghai, China). Bax, Bcl-2 rabbit monoclonal antibodies were purchased from Abcam Biotechnology (Cambridge, UK). Bcl-XL, Bid, Mcl-1 and p53 mouse polyclonal antibody was purchased from Santa Cruz Biotechnology (Santa Cruz, CA, USA). caspase-8, Bak and PUMA antibodies were obtained from Enjing Biotechnology (Nanjing, China) and GAPDH rabbit polyclonal was purchased from Good HERE Biotech Inc. (Hangzhou, China). The MDM2, caspase-3 and caspase-9 rabbit polyclonal antibody and horseradish peroxidase-conjugated secondary antibodies were obtained from Zhongshan Golden Bridge Biotech Inc. (Beijing, China).

##### Cell lines and cell culture

Human prostate cancer cell line (PC-3), human esophageal cancer cell line (EC109, EC9706, KYSE450), Human liver cancer cell line (SMMC-7721), human breast cancer cell line(MCF-7), human gastric cancer cell line (MGC-803), human neuroblastoma cell line(SHSY5Y), human normal liver L-02 cells were purchased from Shanghai Institute of Cell Line Bank. Human gastric cancer cell line (BGC-803) and human liver cancer cell line (ZIP77) were kindly provided by Shanghai Institute of Materia Medica. Human normal esophageal cell line (Het-1A) was *exchanged with* the First Affiliated Hospital of Zhengzhou University. All cells were maintained in RPMI-1640 complete medium with 10% FBS and 100 U/mL penicillin and 100 g/mL streptomycin.

##### Cytotoxic activity determination

The IC_50_ values (concentrations required to inhibit tumor cell proliferation by 50%) for by241 against all human cancer and normal cell lines were determined using the MTT assay. Briefly, cells were trypsinized and incubated at 96-well plates, 24 hours later, cells were treated with 200 μL media containing serial concentration of by241 and cultured for another 72 hours. To detect the NAC effect on by241 induced cytotoxicity, MGC-803 cells were exposed to by241 alone at 6.25 μM, 12.5 μM or by241 pre-incubation with NAC(2.5 mM, 5 mM) for 2 h, followed by 24 h treatment. 20 μL MTT were added to each well and cell were then incubated for 4 hours. Then, the medium was removed and 150 μL DMSO was then added to each well. Absorbance values were measured at 490 nm using an enzyme-link immunosorbent assay reader after shaking for 10 minutes. The cell viability rate was calculated as follows: viability rate = Abs490 treated cells/Abs490 control cells × 100%. The drug concentration required to inhibit cell growth by 50% (IC_50_) was determined from concentration-response curves created with SPSS19.0 software. The results are reported as the mean ± standard deviation (SD) of three independent experiments. The cell viability curves at different concentrations of by241 were created with Graphpad Prism 6.0 software.

##### Clonogenic Assay

MGC-803 cells (500 cells/well) were placed in 6-well plates. After incubation for 24 hours, the media were replaced by fresh media containing different concentration of by241. After 12 days’ treatment, the cells were washed twice with PBS, fixed with 4% paraformaldehyde for 20 minutes, and stained for 30 minutes at 37 °C with crystal violet staining. The clones were imaged under the microscope and the numbers were calculated by Image J software. A group of >50 cells was defined as one colony. The inhibition rate was determined by the number of colonies. Inhibition rate = (1 − number of treatment/number of control) × 100%. All experiments were performed in triplicate.

##### Cell morphology analysis and Hoechest 33258 staining

Cell nuclear apoptotic morphology was analyzed by Hoechst 33258 staining (Sigma, USA). MGC-803 cells or EC9706 cells were seeded at 4 × 10^5^ cells/well in 6-well plates and incubated overnight for adherent and treated with by241 at 2.5 μM, 5 μM or control for 24 hours. The morphological changes were observed under an inverted microscope. Next, cells were harvested and washed twice with PBS, fixed with immunostaining fix solution (Beyotime Institute of Biotechnology, China) for 10 minutes at room temperature and added with 500 μL PBS containing 5 μg/mL Hoechst 33258. The cells were then incubated for additional 30 minutes and carefully washed twice with PBS. Apoptotic cells were examined and identified, according to the condensation and fragmentation nucleus by Nikon inverted fluorescence microscopy.

##### Cellular apoptosis analysis

Cell apoptosis was also quantified by FACS with Annexin V-FITC/PI staining kit from KeyGEN BioTECH (Nanjing, China). According to the manufacturer’s instruction, MGC-803 cells or EC9706 cells were plated in 6-well plates (5.0 × 10^6^ cells/well) and incubated for 24 hours, then cultured with different concentration of by241 for another 12 or 24 hours. Cells were harvested and washed twice with cold PBS and then resuspended in 500 μL binding buffer, containing 5 μL Annexin V-FITC and 5 μL PI, which were then kept in the dark at 37 °C for 30 minutes. Ten thousand cells were collected for flow cytometry analysis (Accuri C6). The apoptotic cells were identified by the localization of Annexin V and PI.

For inhibition increased cleave of caspases-3, 9, we used the pan caspase inhibitor Z-VAD-FMK (APEXBIO). MGC-803 cells were treated pretreatment with Z-VAD-FMK for 1h, followed by241 treatment. 24 hours later, all cells were harvested, stained with Annexin V-FITC/PI, and detected using flow cytometry.

##### Mitochondrial membrane potential (*ΔΨm*) analysis

Mitochondrial membrane potential (*ΔΨm*) was determined by the fluorescent dye JC-1 (Beyotime Institute of Biotechnology, China). Generally, harvested cells were washed twice with PBS and incubated with 10 mg/L JC-1 for 30 min at 37 °C. After incubation with the dye, the cells were collected and carefully washed twice with PBS and then resuspended in 500 μL ice-cold PBS. Flow cytometer was used to measure the fluorescence intensity under an excitation at 488 nm and 535 nm.

##### Intracellular ROS assay

Intracellular ROS generation was detected using DCFH-DA (2,7-dichlorodihydrofluorescein diacetate, Beyotime Institute of Biotechnology, China). Briefly, harvested cells were washed twice by serum-free medium, loaded with 10 μM DCFH-DA and incubated in cell incubator. 30 minutes later, flow cytometry analysis and fluorescence microscope detection were carried out immediately. Ten thousand cells were collected for flow cytometry analysis (Accuri C6). The ROS levels were assayed by the FlowJo software.

##### Western Blot Analysis

MGC-803 cells were cultured at different concentrations of compound by241 for 12 h, both adherent and floating cells were collected with trypsin/EDTA, and then Western blot analysis was performed. The cells were lysed with RIPA cell lysis buffer (1% NP-40, 0.1% sodium dodecyl sulfate (SDS), 150 mM NaCl, 25 mM Tris-HCl, 1% deoxycholic acid sodium salt, 1% PMSF) that contained a protease inhibitor cocktail for 30 min on ice. Total protein were extracted and separated by 6% or 10% SDS-PAGE gel and transferred to a nitrocellulose membrane. The membrane was blocked in Tris-buffered saline (TBS) containing 5% non-fat milk for 2 hours at room temperature and then incubated with the indicated primary antibodies overnight at 4 °C. After that, the membrane was washed three times with TBST and incubated with horseradish-peroxidase-conjugated secondary at room temperature for 2 hours. Then, the membrane preparations were washed three times with TBST and examined by enhanced chemiluminescence. The films were subsequently scanned, and the results were determined using Image J software.

##### Assessment of acute oral toxicity of by241–fixed dose procedure

Animals were treated according to protocols established by the ethics committee of Zhengzhou University and the *in vivo* experiments were carried out in accordance with the approved guidelines and approved by the ethics committee of Zhengzhou University. Acute oral toxicity assessment of by241 was performed according to the fixed dose procedure according to Organization for Economic Cooperation and Development (OECD) Guidelines for the Testing of Chemicals. Groups of 10 mice were dosed in a stepwise procedure using the fixed doses of 1000, 500 and 250 mg/Kg. Oil was used as the control in this experiment. Animals were observed individually at least once during the first 30 minutes after administration, periodically during the first 24 h (with special attention during the first 4 hours) and daily thereafter for a period of 14 days. The observation principally included changes in skin and fur, eyes and mucous membrane and autonomic changes. Food and water were provided throughout the experiment. The animals were weighed each three day and the number of death was noted if applicable.

##### *In vivo* anti-tumor activity

Animals were treated according to protocols established by the ethics committee of Zhengzhou University and the *in vivo* experiments were carried out in accordance with the approved guidelines and approved by the ethics committee of Zhengzhou University. Female BALB/c nude mice (18 g, aged 4-5 weeks) were purchased from Human SJA Laboratory Animal Co. Ltd. (Hunan, China). Mice were subcutaneously implanted with MGC-803 cells (5 × 10^6^ cells per mouse) in the back right flank. Once the volumes reached approximately 100 mm^3^, the mice were randomly divided into corresponding saline (negative control) group, 5-FU (15 mg/Kg, positive control) group, by241 (40 mg/Kg) treatment group, by241 (80 mg/Kg) treatment group and by241 (120 mg/Kg) treatment group (n = 8 mice in each group). The treatment groups received intragastric administration of by241 (40, 80 or 120 mg/Kg) per day for a period of 21 days. 5-FU (15 mg/Kg) was intravenous injection once every three day. After the period, the mice were euthanized and the tumors were isolated and weighed. The body weight was measured and tumor size was determined by vernier caliper measurements every other day to monitor drug potency.

##### Statistical analysis

All data are expressed as the mean ± standard deviation (SD) of three independent biological experiments. Statistical significance was assessed using the two tailed Student’s *t*-test (for comparisons of two treatment groups) and one-way ANOVA (for comparisons of three or more groups). *P < 0.05, ^#^*P* < 0.05 and ** *P* < 0.01, ^##^*P* < 0.01 were considered statistically significant.

## Additional Information

**How to cite this article**: Shi, X.-J. *et al*. Structurally novel steroidal spirooxindole by241 potently inhibits tumor growth mainly through ROS-mediated mechanisms. *Sci. Rep.* **6**, 31607; doi: 10.1038/srep31607 (2016).

## Figures and Tables

**Table 1:** *In vitro* cytotoxicity of by241 against several human cancer cell lines and two human normal cell lines.

	IC_50_ (μM)^a^										
	EC109	EC9706	KYSE450	MGC-803	BGC-803	SMMC-7721	ZIP77	MCF-7	PC-3	Het-1A	L-02
by241	5.62 ± 0.75	4.68 ± 0.67	8.73 ± 0.94	2.77 ± 0.44	1.18 ± 0.71	4.83 ± 0.68	2.71 ± 0.43	6.25 ± 0.80	6.36 ± 0.80	20.15 ± 1.50	21.80 ± 1.34
5-FU	9.31 ± 0.12	8.96 ± 0.33	nd	4.02 ± 0.27	2.23 ± 0.04	2.14 ± 0.25	3.32 ± 0.04	6.93 ± 0.03	15.03 ± 0.96	nd	4.37 ± 0.23

^a^Inhibitory activity was assayed by exposure for 72 h to substances and IC_50_ is the concentration of by241 required to inhibit the cell growth by 50% compared to an untreated control. Data are presented as the means ± SDs of three independent experiments; nd means not determined.

**Table 2:** The acute oral toxicity of by241 at fixed doses.

Dose (mg/Kg)	Survival rate and side effects
250	100% survival rate, no serve toxic effect
500	100% survival rate, no serve toxic effect
1000	100% survival rate, no serve toxic effect
